# Acute Toxicity Evaluation of Lindane-Waste Contaminated Soils Treated by Surfactant-Enhanced ISCO

**DOI:** 10.3390/molecules27248965

**Published:** 2022-12-16

**Authors:** Aurora Santos, Raúl García-Cervilla, Alicia Checa-Fernández, Carmen M. Domínguez, David Lorenzo

**Affiliations:** Departamento de Ingeniería Química y de Materiales, Ingeniería Química y de Materiales, Facultad de Ciencias Químicas, Universidad Complutense Madrid, Ciudad Universitaria S/N, 28040 Madrid, Spain

**Keywords:** ecotoxicity, soil remediation, DNAPL, persulfate, ISCO, S-ISCO, chlorinated organic compounds

## Abstract

The discharge of lindane wastes in unlined landfills causes groundwater and soil pollution worldwide. The liquid waste generated (a mixture of 28 chlorinated organic compounds, COCs) constitutes a dense non-aqueous phase liquid (DNAPL) that is highly persistent. Although in situ chemical oxidation (ISCO) is effective for degrading organic pollutants, the low COCs solubility requires high reaction times. Simultaneous injection of surfactants and oxidants (S-ISCO) is a promising technology to solve the limitation of ISCO treatment. The current work studies the remediation of highly polluted soil (COCs = 3682 mg/kg) obtained at the Sardas landfill (Sabiñáñigo, Spain) by ISCO and S-ISCO treatments. Special attention is paid to acute soil toxicity before and after the soil treatment. Microtox^®^, modified Basic Solid-Phase Test (mBSPT) and adapted Organic Solvent Sample Solubilization Test (aOSSST) were used for this scope. Persulfate (PS, 210 mM) activated by alkali (NaOH, 210 mM) was used in both ISCO and S-ISCO runs. A non-ionic and biodegradable surfactant selected in previous work, Emulse^®^3 (E3, 5, and 10 g/L), was applied in S-ISCO experiments. Runs were performed in soil columns filled with 50 g of polluted soil, with eight pore volumes (Pvs) of the reagents injected and 96 h between successive Pv injections. The total treatment time was 32 days. The results were compared with those corresponding without surfactant (ISCO). After remediation treatments, soils were water-washed, simulating the conditions of groundwater flux in the subsoil. The treatments applied highly reduced soil toxicity (final soil toxicity equivalent to that obtained for non-contaminated soil, mBSPT) and organic extract toxicity (reduction > 95%, aOSSST). Surfactant application did not cause an increase in the toxicity of the treated soil, highlighting its suitability for full-scale applications.

## 1. Introduction

Lindane (γ-hexachlorocyclohexane, γ-HCH) was used as a broad-spectrum pesticide during the second half of the 20th century. It was manufactured by benzene photochlorination, yielding a mixture of isomers (α-, β-, γ-, δ-, ε-HCH) known as technical HCH. γ-HCH is the only isomer with insecticidal properties, and the HCHs mixture requires purification by distillation with solvents and fractional crystallization [1]. This process was highly inefficient, and for each tonne of lindane, approximately 6–10 tonnes of other HCH isomers were generated [2]. Solid and liquid wastes from lindane production were usually dumped in the vicinity of the production sites, resulting in significant soil and groundwater pollution. Due to its danger, the production and use of lindane were banned in most countries. Nevertheless, there are many HCH-polluted spots around the world requiring urgent remedial actions. One particular case is that of Sabiñánigo (Huesca, Spain), where the company INQUINOSA dumped more than 140,000 tonnes of HCH waste in two unfilled landfills: Sardas and Bailín [3].

Liquid waste, produced from failed dechlorination reactions, distillation tails, and solvent residues, constitutes a dense non-aqueous phase liquid (DNAPL) composed of a mixture of 28 chlorinated organic compounds (COCs), including chlorobenzenes, HCHs, and heptachlorocyclohexanes (HeptaCHs) [4], whose hydrophobic character generally increases with the chlorine content of the molecule [5]. It has a density of about 1.5–1.8 g/mL and migrates through the subsurface until it encounters an impermeable layer. In its pathway, DNAPL is adsorbed and entrapped in the soil particle’s pores with small particle sizes, contaminating the subsoil and groundwater.

To solve this issue, in situ chemical oxidation (ISCO) has been successfully applied to remediate COCs-polluted sites in the last decade [4,6,7,8,9,10]. However, DNAPL pollutants are hydrophobic organic compounds (HOCs) with low aqueous solubility. Since oxidation reactions generally occur in the aqueous phase, the efficiency of the ISCO process for the remediation of soils heavily polluted is limited, requiring high reaction times [11,12]. To overcome this limitation, the simultaneous injection of surfactants and oxidants (S-ISCO) has recently attracted attention [11,12,13,14]. The surface-active properties of surfactants enhance the solubilization of HOCs in the aqueous phase and improve their availability for oxidation in the aqueous phase, increasing the oxidation rate in this phase and diminishing the time required for HOCs removal [11,15]. Surfactants are also oxidized, generating finally organic by-products without surfactant capacity [16].

The use of persulfate (PS) as an oxidant has recently gained attention vs. other oxidants commonly used, such as hydrogen peroxide, due to its higher stability in the subsoil and groundwater and relatively low cost [17,18]. PS can be activated in different ways to generate different radical species, i.e., sulfate and hydroxyl radicals, with higher oxidizing power [18,19,20]. In the case of soils with relevant carbonate content and buffered pH, the alkaline activation of PS (pH > 12) is preferable over other types of activation, such as iron at acidic pH. In the alkaline activation of PS at pH > 12, hydroxyl radicals are the main radical species (Equations (1) and (2)) [21,22,23]. ${\mathrm{OH}}_{\;}^{\cdot }$ radical has high oxidative potential (Eo = 2.8 V), the highest of available oxidative agents (only after fluorine). Hydroxyl radicals can oxidize organic molecules that cannot be oxidated by reactive oxygen, ozonation, hydrogen peroxide alone, or chlorine. Moreover, at these alkaline conditions, the compounds with the highest chlorine content of DNAPL from lindane wastes, HCHs and HeptaCHs, are dehydrochlorinated to trichlorobenzenes (TCBs) and tetrachlorobenzenes (TetraCBs), respectively [24,25]. Hydroxyl radicals have shown good effectivity on the abatement of mixtures of chlorobenzene isomers in the aqueous phase [4]. Chlorobenzenes are more soluble in the aqueous phase and more susceptible to being attacked by hydroxyl radicals than the parent pollutants, decreasing the remediation times required [21,23,24,26,27].

(1) $$2S_{2}O_{8}^{2-}+2H_{2}O\overset{{\mathrm{OH}}^{-}\;}{\to }3{\;\mathrm{SO}}_{4}^{2-}+{\mathrm{SO}}_{4}^{-}+O_{2}^{-}+4H^{+}$$

(2) $${\mathrm{SO}}_{4}^{-}+{\;\mathrm{OH}}^{-}\;\to {\;\mathrm{SO}}_{4}^{2-}+{\mathrm{OH}}_{\;}^{\cdot }$$

In this context, a recent study has demonstrated that S-ISCO, which implies the simultaneous injection of a surfactant and an oxidant, was successfully applied in the remediation of real soil highly polluted with lindane wastes [13]. The surfactant addition (S-ISCO, in this case, E-Mulse^®^ 3 as a surfactant and PS activated by alkali as an oxidant) enhances the elimination of COCs compared with the ISCO process, increasing the COCs conversion from 40.1% to 90.4% [13] and reducing the reaction times. In addition to showing high extractive and solubilizing abilities, the surfactants used for S-ISCO applications must be of low ecotoxicity for the soil and biodegradable [5,13,28]. In this line, it must be highlighted that although the use of surfactants in soil remediation has grown in the last years, there is limited information regarding their intrinsic toxicity, especially how their use affects soil toxicity after the S-ISCO treatments, which is decisive for their implementation. Moreover, previous works proved that surfactants react with hydroxyl radicals in competitive reactions with chlorinated organic pollutants [15]. However, surfactants are not mineralized [14], and the toxicity of surfactant by-products remaining in the soil or the aqueous phase must be evaluated.

This work explores the effect of ISCO and S-ISCO treatments on acute soil toxicity using Microtox^®^ bioassays. For this purpose, an alluvial highly contaminated by DNAPL, located in the Sardas landfill (Sabiñanigo, Spain), has been selected. A non-ionic surfactant E3, selected in previous works [13], has been used in S-ISCO column experiments. This surfactant was relatively stable against the oxidant (PS activated by alkali) with good solubilization of COCs trapped in the soil [15,29]. The acute toxicity of the initial and polluted soil after ISCO and S-ISCO treatments, as well as that of non-polluted soil, has been determined. Two Microtox^®^ procedures were recently validated and optimized to measure the acute toxicity of these soils: the modified Basic Solid-Phase Test (mBSPT) and the adapted Organic Solvent Sample Solubilization Test (aOSSST). From our knowledge, it is the first study that focuses on evaluating the toxicity of soils after ISCO and S-ISCO remediation treatments, including the effect of soil alkalinization and surfactants addition, giving practical information for a full-scale application.

## 2. Results and Discussion

### 2.1. Soil and DNAPL Characterization

The physicochemical characteristics of the polluted soil have been determined following the procedures detailed in Section 3.4. The mineral composition of the soil was as follows: carbonates (453 g/kg), iron (32.6 g/kg), magnesium (7.4 g/kg), calcium (182.3 g/kg), manganese (0.8 g/kg), sodium (1.7 g/kg), aluminum (15.9 g/kg) and potassium (3.2 mg/kg). The high carbonate content (given as calcium carbonate) produces strongly buffered soils (pH = 7.5). TOC and IC of the polluted soil were 0.1 and 5.4%, respectively, in agreement with the values previously reported [13,30]. The total COCs concentration in the polluted soil (B2) was 3682 mg/kg, corresponding to a TOC concentration of 910 mg/kg. Therefore, the carbon from the identified COCs in the polluted soil fit well with the measured TOC, and the presence of other organic compounds in significant amounts can be neglected. The COCs distribution in soils B1 and B2 are shown in Table 1.

**Figure 1:**
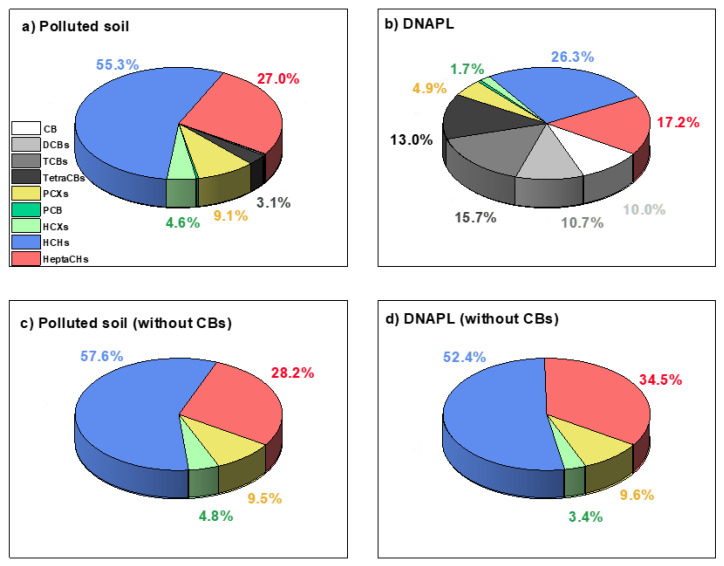
COCs distribution (grouped by families) in the polluted soil B2 (C_COCs,0_ = 3682 mg/kg) (**a**), DNAPL (**b**), polluted soil without CBs (**c**), and DNAPL without CBs (**d**) (mass percentages).

DNAPL from the landfill was also analyzed, and its composition is shown in Table 1. The mass distribution of COCs in the polluted soil (B2) and the DNAPL (source of soil contamination) are summarized in Figure 1a,b, respectively. COCs isomers families have been grouped to facilitate the interpretation of the results: chlorobenzene (CB), dichlorobenzenes (DCBs), trichlorobenzenes (TCBs), tetrachlorobenzenes (TetraCBs), pentachlorocyclohexenes (PCXs), hexachlorocyclohexanes (HCXs), hexachlorocyclohexanes (HCHs), and heptachlorocyclohexanes (HeptaCHs). As shown, the percentage of chlorobenzenes in the DNAPL (49.5%) is considerably higher than in the polluted soil (3.6%), with HCHs (55.3%) and HeptaCHs (27.0%) being the main soil contaminants. This is explained by the higher volatility of chlorobenzenes [31], causing their loss during storage, sieving, and drying of soil samples [30]. If chlorobenzenes are not considered (Figure 1c,d), DNAPL and polluted soil show almost the same COCs distribution, confirming that this dense phase is the cause of the alluvial pollution. The unpolluted soil (B1, reference) has also been characterized, presenting equivalent physico–chemical characteristics and mineral composition to the polluted soil but with a significantly lower COCs concentration (20.4 mg/kg).

### 2.2. Toxicity of Elutriates from Soil-Aqueous Phase Contact

Soils B1 and B2 were in contact with aqueous phases at neutral pH and a ratio of *V_L_*/*W* = 2 L/kg. After the separation of phases by centrifugation, the aqueous phase composition was analyzed, and the results are shown in Table 2. The ${\mathrm{EC}}_{50}{}_{j}$ value of each COC is also included in Table 2 as $\left(\frac{\mathrm{mg}}{L}\right)$ [30,32]. About half of the COCs in the aqueous phase are non-commercial or have unknown ${\mathrm{EC}}_{50}{}_{j}$ values. The toxicity of the aqueous phases has been experimentally determined by Equation (6) (TU exp) and compared with those estimated by Equation (8) (TU estim). Both values are also included in Table 2. As can be seen, the experimental TUs values are higher than the estimated ones. This difference can be explained considering that estimated TUs by Equation (8) is undervalued because of the unknown ${\mathrm{EC}}_{50}{}_{j}$ values of some compounds included in Table 2.

The composition of the aqueous Pv flushed from the soil column after the alkali pretreatment is also included in Table 2. As shown, HCHs-PentaCXs and HexaCXs-HeptaCHs are not present in this alkaline effluent, in agreement with dehydrochlorination of these compounds to TCBs and TetraCBs, respectively, which was previously described in the literature [23,25]. The toxicity was measured after the neutralization of this alkaline column effluent. The estimated (Equation (8)) and measured TUs (Equation (6)) are also shown in Table 2. The ${\mathrm{EC}}_{50}{}_{j}$ values of all the COCs present in the aqueous phase at alkaline conditions are known, which explains the similar values obtained between experimental and estimated TUs in this case. It should be highlighted that COCs removed from the soil after contact with the aqueous phase were a small fraction of the initial COCs in the soil. About 3% and 2% of COCs in soil B2 are lixiviated when 1 L of the aqueous phase is in contact with 1 kg of soil B2 at neutral and alkaline pH, respectively.

### 2.3. ISCO and S-ISCO Experiments

The remaining PS was analyzed at each Pv flushed with the next Pvs injected in T1 (ISCO, without surfactant), T2 (S-ISCO, 5 g/L of surfactant), and T3 (S-ISCO, 10 g/L of surfactant) and the values obtained are shown in Table S1 (Supplementary Material). The pH value (not shown) was always higher than 12. As shown in Table S1, a significant concentration of PS remains unreacted at each Pv flushed. The lower the surfactant concentration injected, the higher the remaining PS in the Pv flushed. This fact is explained by the unproductive consumption of PS caused by surfactant oxidation [29]. The Pvs flushed from the different columns were gathered, allowing the oxidation reaction continues in the aqueous phase, which simulates the progress of the aqueous phase in the subsoil. PS, COCs, and surfactant concentration were measured in the 8 gathered Pvs 96 h after the last Pv was eluted. The composition of these aqueous phases is shown in Table S2. Thiosulfate was added to eliminate the remaining PS, and pH was neutralized by adding sulfuric acid. The toxicity of gathered Pvs eluted from T1, T2, and T3 columns were experimentally measured (Equation (6)). Moreover, TUs have been estimated (Equation (8)) using the COCs concentration included in Table S2. Similar values were obtained for experimental and estimated TUs, indicating that no other toxic compounds were present in the aqueous phases.

Each soil column was washed with 8 Pvs of tap water 96 h after the last Pv with the reagents was injected, as described in the experimental section. The Pv flushed with each Pv injected was collected, and the gathered aqueous phases were analyzed. Finally, the soil columns were disassembled, dried, homogenized, and analyzed. The remaining COCs concentration measured in the soil of columns T1, T2, and T3 is shown in Table 3.

A mass balance of COCs has been accomplished considering the initial mass of COCs in soil B2 ($w_{{\mathrm{COC}}_{s}\;initial\;in\;soil})$, the mass of COCs recovered in the treated soil ($w_{{\mathrm{COC}}_{s}\;final\;in\;soil})$, the mass of COCs in flushed Pvs from the columns T1, T2, and T3 containing PS ($w_{{\mathrm{COCs}}_{P_{v\;PS}}})$ and the mass of COCs in the flushed Pvs of tap water ($w_{\mathrm{COCs}}{}_{P_{TW}})$. The results obtained are shown in Table 4. The removal of COCs by oxidation was calculated as follows:

(3) $$X_{\mathrm{COCs}}=1-\frac{w_{{\mathrm{COC}}_{s}\;final\;in\;soil}+w_{{\mathrm{COCs}}_{P_{v\;PS}}}+w_{\mathrm{COCs}}{}_{P_{v_{TW}}}}{w_{{\mathrm{COC}}_{s}\;initial\;in\;soil}}$$

The removal of the COCs mixture obtained from the same landfill and soil was studied in previous works by ISCO and S-ISCO in batch and column operation [13]. Column study was carried out with a lower number of pore volumes injected (4) and a lower reaction time of the oxidant in the column (15 days). In that work, the column runs were carried out with a lower number of pore volumes injected (4) and a lower reaction time of the oxidant in the column (15 days in total). Therefore, a lower COCs conversion was found in that work (from 0.5 to 0.7) than the COCs conversion obtained in this work (from 0.9 to 0.97). García-Cervilla et al. [13] found COCs conversion in ISCO treatment was always lower than in S-ISCO, as noticed in this work. The COCs conversion improvement by the surfactant addition was also noticed in the oxidation of the same DNAPL used in this work with PS activated by alkali in the absence of soil [16]. The improvement of surfactant addition was also reported with other NAPLs and soils [11,14]. As an example, Lominchar et al. [14] found a TPH conversion in real polluted soil of about 65% and 95% after 25 days in the presence or absence of surfactant (E3) using persulfate activated by alkali as the oxidant.

### 2.4. Microtox^®^ Toxicity Evaluation

**Figure 2:**
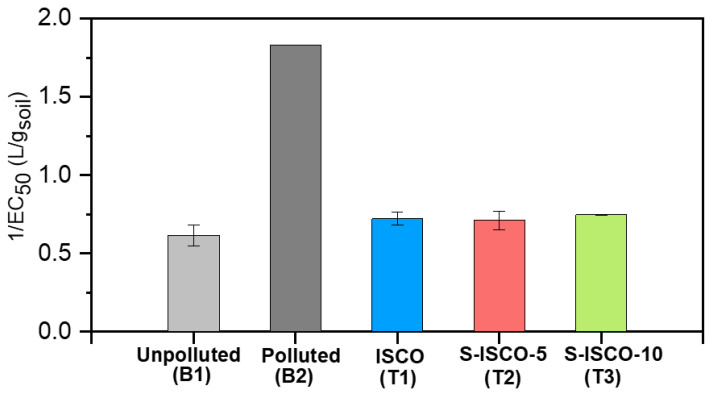
Soil toxicity (1/EC_50,_ Microtox^®^ mBSPT) of the unpolluted, polluted, and treated soils.

The acute toxicity of the unpolluted (B1), polluted (B2), and treated soils (ISCO, S-ISCO-5, and S-ISCO-10) was determined by the modified Basic Solid-Phase Test (mBSPT). The results of soil toxicity (expressed as 1/EC_50_, L/g_soil,_ Microtox^®^ mBSPT) are represented in Figure 2. Kwan and Dutka [33] proposed the following soil toxicity classification: EC_50_ > 10 g/L nontoxic, 5 g/L < EC_50_ ≤ 10 g/L moderately toxic, and EC_50_ ≤ 5 g/L very toxic. Thus, according to this classification, the polluted soil studied (EC_50_ = 0.55 g/L, Figure 2) is considered highly toxic. The fact that the unpolluted soil (considered as reference), with low COCs concentration (20.4 mg/kg _soil_), exhibits intrinsic toxicity (EC_50_ = 1.64 g/L, Figure 2) would indicate that there are toxic compounds for the bacteria in the soil matrix [30]. On the other hand, as previously mentioned, estimated and measured TUs of the aqueous phases are in good agreement, indicating that no other toxic compounds than COCs are in the aqueous phase extracted from the soil. Therefore, the intrinsic soil toxicity corresponds to compounds in soil that do not pass to the aqueous phase.

The toxicity of soils treated by ISCO and S-ISCO and further washing post-treatments decreased significantly, reaching toxicity values equivalent to that of the unpolluted soil (Figure 2). No differences were found between the toxicity values obtained for ISCO and S-ISCO experiments with the surfactant concentration range studied. High COCs conversion (≥90%) was obtained in all these runs due to the high number of Pvs injected. Therefore, it can be concluded that neither the surfactant application (at the conditions tested) nor its concentration generates toxicity in the treated soil.

**Figure 3:**
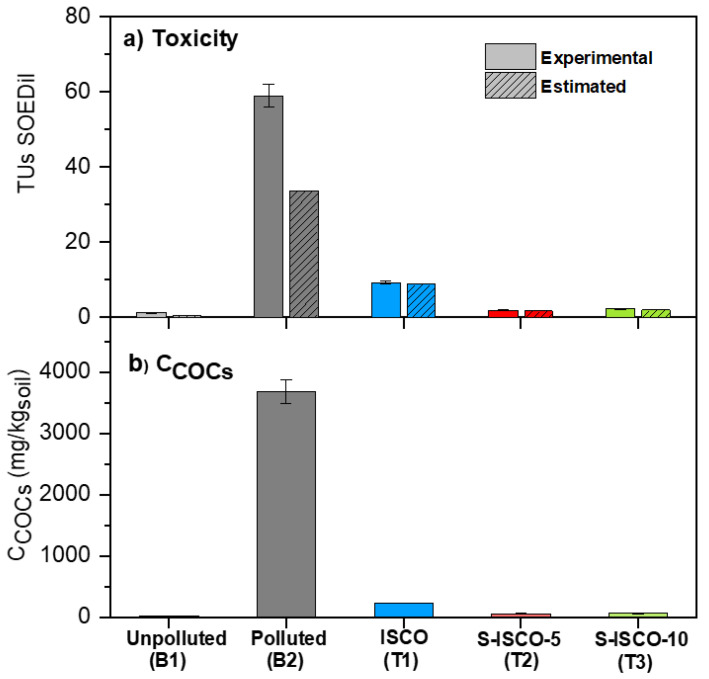
Soil organic extract toxicity (TUs SOEDil, Microtox^®^ aOSSST) (**a**) and COCs concentration (mg/kg_soil_) (**b**) of the unpolluted, polluted, and treated soils.

As explained in the experimental section, the SOE phase was obtained after extracting COCs from 15 g of soil with 15 g of MeOH. Diluted Soil Organic Extract, SOEDil corresponded to 3.2% of SOE and 96.8% of diluent (mass percentages). The IC_50_ values of SOEDil were obtained for the five soils under study: B1 (unpolluted), B2 (polluted), and soils recovered from the column after ISCO, S-ISCO-5, and S-ISCO-10 treatments. COCs concentrations in SOE (as mg/kg MeOH) were measured, obtaining similar values to COCs concentration in soil (as mg/kg) because about 98% of COCs in soil were extracted, and the mass ratio MeOH/soil used was 1:1. Accordingly, COCs concentration in SOEDil phase was 3.2% of COCs concentration in SOE. Experimental TUs of the SOEDil phase were calculated (Equation (6)) and estimated (Equation (8)), and the values obtained are shown in Figure 3a. As can be seen, the trend obtained for soil toxicity reduction was similar to that above shown for the mBSPT results. In this case, the differences observed between polluted and unpolluted soil toxicity are greater in the SOEDil phase. This finding can be explained as most of the COCs in soil are in the SOE extract.

On the contrary, due to its hydrophobic character, most of the COCs mass remains in the soil phase when soil is in contact with the aqueous phase (mBSPT method). Moreover, the experimental and estimated TUs of SOEDil (aOSSST method) are similar in soils from ISCO, S-ISCO-5, and S-ISCO-10, indicating that non-other toxic compounds than identified COCs remain in the treated soils. On the other hand, experimental TUs in SOEDil are higher than estimated TUs in original soil B2 (Figure 3a). These lower TUs values can be explained as some COCs in the organic extract have unknown EC_50_ values. As the aOSSST measures the toxicity of the compounds extracted in the organic phase (methanol), the concentration of COCs in the different soils’ organic extracts is represented (Figure 3b) to confirm the proportionality between both measures. Comparing Figure 3a,b, it is demonstrated that the toxicity of the organic extracts correlates well with the COCs concentration in soil, as demonstrated in previous work [30].

The toxicity of the organic extracts of the treated soils decreases greatly (including the percentage of the initial one), reaching values equivalent to those of the reference soil B1. Finally, as previously mentioned for the case of soil toxicity, the aOSSST confirms that the addition of surfactant in the range studied (5–10 g/L) does not cause an increase in soil toxicity (S-ISCO vs. ISCO experiments). Thus, either of the proposed in situ treatments (ISCO or S-ISCO, with alkaline activation of PS) can be proposed for a full-scale application, as they lead to a high COCs reduction and restore the soil to its original toxicity value.

## 3. Materials and Methods

### 3.1. Landfill Soil Samples

The polluted soil (B2) was supplied by the company EMGRISA from a borehole drilled at the alluvium from one of the most contaminated areas in Sardas landfill (Sabiñánigo, Spain) [15,23]. The soil layer used was located at a depth of 15.6–16.1 m below ground level, where the groundwater flows, and it is mainly composed of gravel and sand with some clay interbedded. Different contamination levels were noticed depending on the depth and the soil particle size [23]. The soil sample from the alluvium was dried at room temperature (22 ± 2 °C) and sieved in different fractions using an electromagnetic sieve shaker (BA-200-N). The soil particles higher than 2 mm were rejected, and the fraction with a particle size between 2–0.25 mm was selected to carry out the current study. Liquid wastes from lindane production dumped decades ago caused the contamination of soil and groundwater in the Sardas landfill [4,23].

A reference soil sample from the alluvial with a significantly lower COCs concentration (20.4 mg/kg) (unpolluted soil, B1) was obtained at the same depth as the previous one but outside the perimeter of the landfill-contaminated area. This soil sample was handled as the polluted soil (dried and sieved). It was used to eliminate physical interferences of soil (soil matrix such as metals, organic matter, etc.) from the toxic effects due to the pollutant’s presence in the toxicity analysis [30,34]. Moreover, DNAPL in contact with the non-permeable marls layer, which caused groundwater pollution, was extracted at the bottom of the alluvial.

### 3.2. Chemicals

The bacterial reagent (*Vibrio fischeri*, strain NRRL B-11177) and the reconstituent solution used in the Microtox^®^ acute toxicity bioassays were provided by ModernWater Inc (New Castle, DE, USA). Sodium chloride (NaCl), used to prepare diluent and osmotic adjustment solutions, was supplied by Sigma-Aldrich. Phenol, taken as a standard to test the performance of the Microtox^®^ system, was provided by Riedel-de Haën. Methanol (CH_3_OH), used as the solvent for the Organic Solvent Sample Solubilization Test, was provided by Fisher.

Commercial COCs of analytical quality were used to prepare the standards solutions for the calibration curves: chlorobenzene (CB), dichlorobenzene isomers (DCBs), trichlorobenzene isomers (TCBs), tetrachlorobenzene isomers (TetraCBs), and hexachlorocyclohexane isomers (HCHs), were provided by Sigma Aldrich. Bicyclohexyl (C_12_H_22_) and tetrachloroethane (C_2_H_2_Cl_4_), used as internal standards (ISTD) for COCs quantification, were also purchased from Sigma-Aldrich. Methanol and n-hexane (C_6_H_14_, Honeywell) were used to extract COCs from the soil and the aqueous phase, respectively.

Sodium Persulfate (PS) and sodium hydroxide (NaOH) used in ISCO and S-ISCO experiments as the oxidant and the activator, respectively, were provided by Sigma-Aldrich and Fisher. Sodium thiosulfate pentahydrate, provided by Sigma-Aldrich, was used to stop the oxidation reactions. The non-ionic commercial surfactant, E-Mulse^®^3 (E3, Ethicalchem, South Winsor, CT, USA), selected in previous work [13] and provided by EthicalChem, was used for S-ISCO experiments. This surfactant showed high COCs solubilization from the soil and relatively high stability against the oxidant selected (persulfate activated by alkali) [15,29]. All the reagents used in the current work were of analytical quality. A deionizing system (Milli-Q^®^ Direct 8, supplied by Merck, Darmstadt, Germany) was used to obtain Milli-Q water (>18 MΩ cm) for preparing all the aqueous solutions.

### 3.3. ISCO and S-ISCO Experiments

**Figure 4:**
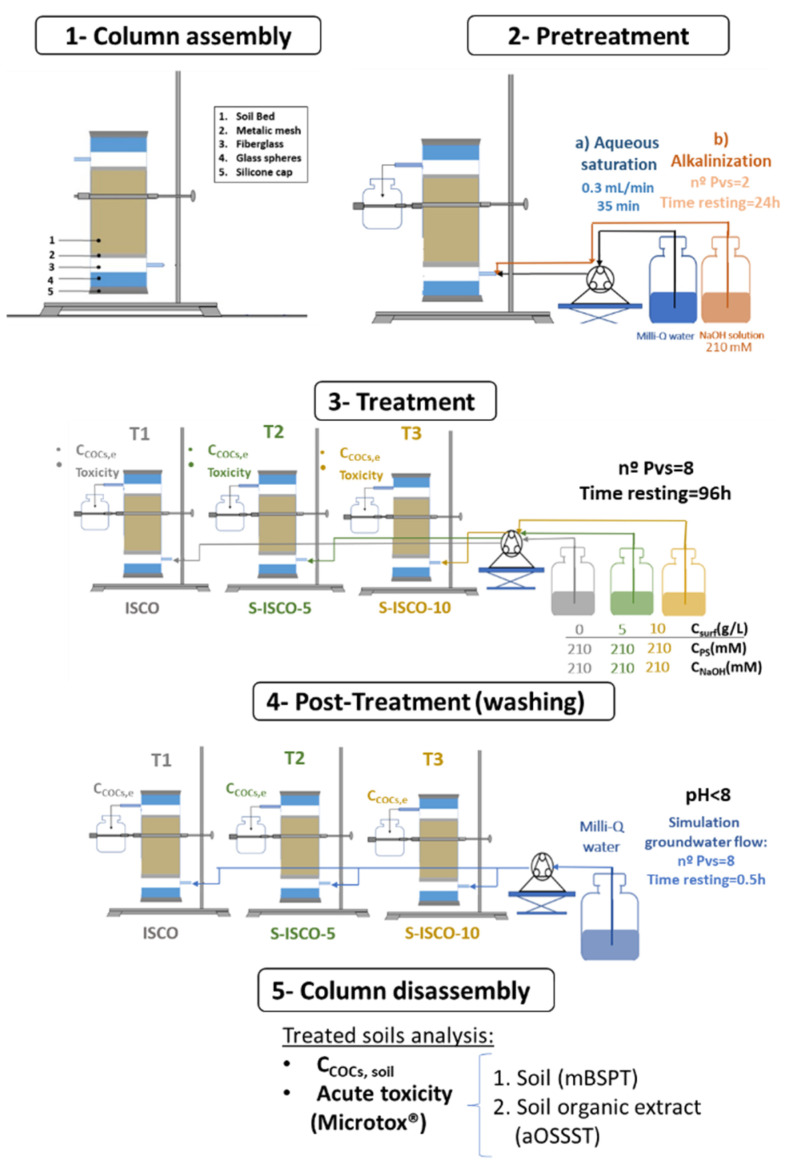
Scheme of the different stages followed in the remediation experiments. Experimental conditions are summarized in Table 5.

The remediation experiments were carried out in glass columns (diameter = 3 cm) with two side ports (4.7 cm between the two side ports). The column characteristics have been summarized in Table 5, and a scheme of the column assembly is shown in Figure 4. The bottom of the column was sealed with a silicone cap. Glass spheres (0.25 mm diameter) were placed at the column bottom, and glass fiber (covering the side port) and metallic mesh, were placed over them. The column was then filled with approximately 50 g of polluted soil (3682 mg/kg of COCs, 0.25–2 mm particle size), and the column assembly was completed by placing other metallic mesh, glass fiber, glass spheres, and the cap (in this order). Three columns were prepared: one for ISCO treatment and two for S-ISCO treatments.

Firstly, one pore volume (Pv) of Milli-Q water was injected into the columns to achieve pore water saturation. The Milli-Q water was injected at 0.3 mL/min (35 min approx.) using a peristaltic pump (Spetec Perimax 12). Then, a new Pv was injected with a NaOH solution (210 mM). After 24 h, the alkaline injection was repeated. The stoichiometric concentration of NaOH for the conversion of all non-aromatic compounds to TCB and TetraCB by dehydrochlorination [4] is 34.2 ${\mathrm{mmol}}_{\mathrm{NaOH}}/{\mathrm{kg}}_{\mathrm{soil}}$ (this value is lower than one Pv injected).

After the alkaline pretreatment, a total of 8 Pvs (containing the desired concentration of oxidant/activator/surfactant, see Table 5) were successively injected into each column (0.3 mL/min) with a time elapsed between each pore volume injection of 96 h. The total time the oxidant injected in the successive pore volumes remained in the column was approximately 32 days. As can be seen in Table 5, the ISCO run (column T1) used an oxidant/activator solution (oxidant and NaOH concentration of 210 mM) without surfactant. The same oxidant and activator concentration were used in the two S-ISCO experiments. The surfactant E3 (5 and 10 g/L) was fed to the columns (T2 and T3, respectively). Operational conditions were selected from previous work [13]. The experiments were performed at room temperature (22 ± 2 °C). The aqueous phase in the columns was flushed with each Pv injected, and the concentration of PS eluted in the Pv flushed was measured. Pvs eluted from the columns were collected and stored. PS, COCs, pH, and surfactant concentration were measured in the gathered aqueous phase. Thiosulfate was added and pH neutralized before the toxicity of the gathered aqueous phase was determined.

After the 8 Pvs with the reagents were injected, 8 Pvs of tap water were successively injected in the columns. Each Pv of tap water remains 30 min in the column before the next Pv of tap water replaces it. The effluents were collected and analyzed. The injection of tap water simulates the groundwater flows in subsoil after the chemical treatment. Finally, the columns were disassembled, homogenized, and dried at room temperature before analyzing the COCs and toxicity of the treated soils.

### 3.4. Analysis

The soil was analyzed before and after applying ISCO and S-ISCO treatments. The received polluted soil (moisture content of around 15% (vol.)) and the treated soils were dried at room temperature (48 h, 22  ±  2 °C). The extraction of COCs from soil was performed by mixing 15 g of the dry soil sample with 30 mL of methanol in 40 mL-PTFE vials at 45 °C in an ultrasound bath (Power sonic 505) for 180 min [21,22,24,30]. Afterthought, the PTFE vials were centrifuged (900 rpm, 10 min, MEDTRONIC-BL-S, JP SELECTA^®^, Barcelona, Spain), and the organic phase was separated from the soil by decantation. COCs concentration was determined by analyzing the organic phase by gas chromatography (GC, Agilent 6890) coupled with flame ionization (CG-FID) and electron capture detectors (CG-ECD) (Agilent 6890) using an HP-5-MS column (HP-5-MS, 30 m × 0.25 mm i.d., 5% phenyl methyl siloxane). COCs identification was previously performed by gas chromatography coupled with a mass spectrometry detector (GC-MSD) [4,22,23]. Bicyclohexyl and tetrachloroethane were added to the organic samples as internal standards (ISTDs) to minimize experimental errors in COCs quantification by GC-FID and GC-ECD, respectively. COCs concentration in the aqueous phase collected at the outlet of the columns was analyzed. In the absence of surfactant (E3), COCs from the aqueous phase were extracted with an organic solvent (hexane, 1:1 mass ratio), and the COCs in the organic phase were analyzed by GC-FID/ECD. In the presence of E3, COCs were analyzed directly after diluting the sample with MeOH (volume ratio 1:10). The GC analyses for both soil and aqueous phases were duplicated. Differences were lower than 5%.

The surfactant concentration in the aqueous phase of the column effluent was determined as equivalent surfactant concentration (*ESC*) [29]. In a glass vial, a volume of the aqueous sample was added. Then, a mass of a synthetic DNAPL with the same composition as those obtained under alkaline conditions was added. Once the chlorobenzenes were solubilized, the emulsion was centrifuged. Finally, the supernatant was analyzed by GC-FID/ECD. The ESC value was calculated by Equation (4):

(4) $$ESC=\frac{\sum COC_{j}{}_{solubilized}}{WSR}$$

*ESC* (g/L) is the surfactant concentration with the same solubilization capacity that the aqueous surfactant solution analyzed. The solubilization capacity of E3 is represented by the mass solubilization ratio (*WSR*) (mg/g). *WSR* of E3 for a mixture of chlorobenzenes under alkaline conditions (1005 mg/g) was obtained elsewhere [35].

The pH of soil and effluent samples was determined using a Basic 20-CRISON pH electrode. In the case of soil samples, pH was measured from a soil-water suspension using an aqueous phase/soil ratio equal to 2. The total carbon (TC) and inorganic carbon (IC) content of the soil sample were determined using a Shimadzu TOC-V CSH analyzer. TC was determined by oxidative combustion (680 °C) using an infrared detector equipped with an SSM-5000A solid sampler (EN 15936:2012). The sample was acidified with phosphoric acid (35%) at 200 °C for IC quantification. Total organic carbon (TOC) was calculated as the difference between TC and IC content. Acid digestion and measurement with a microwave plasma atomic emission spectrometer (4100 MP-AES, Agilent) determined metal cation concentrations in the soil.

### 3.5. Microtox^®^ Toxicity Bioassays

Toxicity of soils (unpolluted, polluted, and treated soils), toxicity of aqueous phases after batch contacting between water and soil (ratio *V_L_*/*W* = 2 L/kg) and toxicity of the column effluents from the different treatments (ISCO, S-ISCO-5, and S-ISCO-10) were determined by the Microtox^®^ bioassay, based on the measurement of the natural luminescence emitted by the marine bacterium *Vibrio fischeri*. Exposure of these bacteria to toxic samples disrupts the respiratory processes, reducing the emitted light, and directly correlating with sampling toxicity [36]. Toxicity analyses were carried out in triplicate using a Microtox^®^ M500 Analyzer (Microbics Corporation, Carlsbad, CA, USA). An internal quality control test using phenol as a reference pollutant was run periodically. The quality of the data obtained in a particular test was evaluated according to the guideline of Doe, et al. [37]. If any of the following conditions were not fulfilled, the test was considered not valid: (i) the light loss should decrease monotonously as the test concentration decreases, (ii) the coefficient of variation of the light readings for the control solutions must be <12%, and (iii) the coefficient of determination (R^2^) must be higher than 0.9. As the Microtox^®^ bioassay was used to measure the acute toxicity of soils, soil organic extracts and effluents, different sample preparation procedures and standard protocols have been employed.

The toxicity of aqueous phases was measured by the Microtox^®^ Basic Test, following the standard operating procedure Microbics [38]. The osmotic control was reached by adding 250 μL of an osmotic adjustment solution (NaCl, 22%) to 2.5 mL of the initial sample. For each sample, four dilutions and a blank were analyzed. Vials with the bacteria reagent were prepared by mixing 0.5 mL of the diluent (NaCl, 2%) and 10 μL of the reconstituted bacteria reagent. After that, 0.5 mL of each diluted sample was added to the next vial, achieving a concentration of 50% of the previous one with each successive dilution (maximum sample concentration of 45%). The bioluminescence inhibition of *Vibrio fischeri* was measured at 15 min of exposure. ${\mathrm{IC}}_{50}$ is the sample dilution ratio that yields a 50% reduction of bacteria light emission calculated by Equation (5):

(5) $${\mathrm{IC}}_{50}\left(\%\right)=\frac{V_{sample}}{V_{total}}100$$

where $V_{sample}$ is the volume of the polluted sample and $V_{total}$ is the volume sum of the sample volume and the added volume of the diluent and the bacteria.

Toxicity units of the aqueous phase (*TUs*) are calculated with Equation (6) [39]

(6) $$TUs=\frac{100}{{\mathrm{IC}}_{50}\left(\%\right)}$$

The effective nominal concentration of a compound (EC_50_, mg/L) is the concentration value in the aqueous phase that reduces light emission intensity by 50%, as indicated by Equation (7).

(7) $${\mathrm{EC}}_{50}j_{\;}\left(\frac{mg}{L}\right)=\frac{C_{j}}{TUs}=\frac{{\mathrm{IC}}_{50}\left(\%\right)}{100}\cdot C_{j}$$

The TUs of a mixture of known composition and ${\mathrm{EC}}_{50}{}_{j}$ values can be estimated with Equation (8) [39]:

(8) $$TUs=\sum \frac{C_{j}}{{\mathrm{EC}}_{50}{}_{j}}$$

where $C_{j}$ is the concentration of each compound in the aqueous phase (mg/L), and ${\mathrm{EC}}_{50\;j}\;$the corresponding effective nominal concentration (mg/L). *TUs* estimated by Equation (8) can be higher or lower than those obtained by Equation (6) due to synergistic (positive or negative effects) or to the presence of non-identified compounds or unknown ${\mathrm{EC}}_{50\;j}\;$values [40,41,42].

Soil samples were crushed and sieved to a particle diameter of less than 0.25 mm before carrying out the Microtox^®^ bioassays. Two different sample preparation procedures and standard protocols were performed to assess the soil toxicity (unpolluted, polluted, and treated). Firstly, the soil toxicity was evaluated by applying the modified Basic Solid-Phase Test (mBSPT) in which an aqueous soil suspension (slurry) is analyzed [30,43]. Slurries were prepared by mixing 10 min 7 g of soil and 35 mL of a diluent (saline solution NaCl 2%), the soil concentration in the slurry was 200 g/L. For each soil sample, the bioluminescent bacteria’s light emitted (I) was measured without filtration for 9 successive dilutions (50% of the previous one) and 3 blanks. The bioluminescence inhibition of the marine bacterium was measured after 30 min of sample exposure. The detailed procedure used in the mBSPT analysis can be found elsewhere [30]. The toxicity results obtained have been expressed as EC_50_ (in *g_soil_/L*), indicating the concentration of soil that yields 50% of light inhibition, as shown in Equation (9).

(9) $${\mathrm{EC}}_{50}\left(g_{soil}/L\right)=\frac{mass_{soil}}{V_{total}}$$

The pH of the samples is crucial for toxicity analysis since the bacterial reagent is sensitive to pH (the effect is minimal between 6.0 and 8.0 Microbics [38]). After the post-treatment (washing with water the soil in the columns), no pH adjustment was required as all samples were within the acceptable range (pH = 6–8).

Most COCs in the soils under study are hydrophobic organic compounds (HOCs), poorly soluble in the aqueous phase, limiting their bioavailability for soil toxicity determination. This limitation was overcome by applying the adapted Organic Solvent Sample Solubilization Test (aOSSST) to soil samples. This test can dissolve organic solvent-soluble compounds from soils, ensuring the total bioavailability of pollutants [44]. Methanol has been chosen as the solvent, as it is also effective in extracting COCs from these soils [21,22,45]. For COCs extraction, 15 g of methanol was placed in contact with 15 g of soil and vials were introduced in an ultrasound bath (Power sonic 505) for 180 min at 45 °C. The vials were cooled (room temperature) and centrifuged (10 min, 9000 rpm, MEDTRONIC-BL-S, JP SELECTA^®^), the supernatant organic phase constituting the soil organic extract (SOE). The methanol maximum allowable concentration (MAC) in the bioassays was 4% in volume, ensuring a null effect of the solvent on the measured toxicity [30]. Therefore, the soil organic extract (SOE) was diluted with a diluent solution (NaCl 2%) to reach a concentration of 4% in the volume of SOE in the total volume of the liquid phase. The resulting liquid phase (SOEDil) contains 3.2% SOE and 96.8% in mass of diluent. IC_50_ and TUs of this liquid phase (SOEDil) were measured as explained before (Equations (5) and (6)). Further information about the aOSSST analysis can be found elsewhere [30]. The estimated TUs of the SOEDil phase can be calculated from the composition of this phase, according to Equation (8).

## 4. Conclusions

This work evaluated the toxicity of the simultaneous addition of a non-ionic surfactant (E3) and an oxidant (PS-alkali activated) in the remediation of real soil highly polluted by lindane wastes (C_COCs_ = 3682 mg/kg). E3 was selected for S-ISCO experiments due to its higher COCs solubilization capacity, lower unproductive oxidant consumption, and higher biodegradability vs. other commercial surfactants. The initial polluted soil showed high acute toxicity, and the toxicity of the soils treated by ISCO and S-ISCO decreased significantly. EC_50_ values of treated soils are equivalent to that obtained for the non-contaminated soil (mBSPT), and TUs of soil organic extract (aOSSST) of treated soils are orders of magnitude lower than TUs of soil organic extract from initial polluted soil. In addition, it has been demonstrated that neither the application of E3 nor its concentration (within the concentration range studied) generates toxicity in the treated soil. Thus, either of the proposed in situ treatments (ISCO or S-ISCO, with alkaline activation of PS) is suitable for a full-scale application, as they lead to a high COCs abatement (>90%) and restore the soil to its original toxicity value.

## Figures and Tables

**Table 1:** COCs composition of DNAPL and soil samples.

COMPOUND		M (g/mol)	C_DNAPL_ (mg/kg)	%w _DNAPL_	C_B1_ (mg/kg)	%w B1	C_B2_ (mg/kg)	%w B2
Name	Acronym							
Chlorobenzene	CB	112.5	96,610.1	10.0	0.00	0.00	0.00	0.00
1,3-dichlorobenzene	1,3-DCB	147.0	5954.1	0.6	0.00	0.00	0.05	0.00
1,4-dichlorobenzene	1,4-DCB	147.0	54,140.5	5.6	0.00	0.00	0.00	0.00
1,2-dichlorobenzene	1,2-DCB	147.0	43,743.0	4.5	0.00	0.00	4.84	0.13
1,3,5-trichlorobenzene	1,3,5-TCB	181.5	1812.8	0.2	0.00	0.00	0.50	0.01
1,2,4-trichlorobenzene	1,2,4-TCB	181.5	129,035.3	13.4	2.00	9.82	7.97	0.22
1,2,3-trichlorobenzene	1,2,3-TCB	181.5	20,061.7	2.1	0.30	1.47	5.72	0.16
1,2,4,5 and 1,2,3,5-tetrachlorobenzene	TetraCB-a	216.0	52,347.6	5.4	2.00	9.82	32.95	0.89
1,2,3,4-tetrachlorobenzene	TetraCB-b	216.0	72,957.8	7.6	4.00	19.64	79.87	2.17
γ-Pentachlorocyclohexene	γ-PentaCX	254.0	21,236.8	2.2	1.80	8.84	80.37	2.18
1,2,3,4,5-Pentachlorobenzene	PCB	250.0	5642.4	0.6	0.30	1.47	13.69	0.37
δ-Pentachlorocyclohexene	δ-PentaCX	254.0	19,435.2	2.0	3.50	17.18	194.26	5.28
θ-Pentachlorocyclohexene	θ-PentaCX	254.0	1548.0	0.2	0.00	0.00	16.35	0.44
Hexachlorocyclohexene-a	HexaCX-a	289.0	6762.4	0.7	0.33	1.62	51.26	1.39
β-Pentachlorocyclohexene	β-PentaCX	254.0	2612.6	0.3	0.00	0.00	43.59	1.18
η-Pentachlorocyclohexene	η-Penta CX	254.0	1612.2	0.2	0.03	0.15	2.28	0.06
Hexachlorocyclohexene-b	HexaCX-b	289.0	2375.3	0.2	0.00	0.00	10.32	0.28
Hexachlorocyclohexene-c	HexaCX-c	289.0	7274.5	0.8	0.00	0.00	106.87	2.90
α-Hexachlorocyclohexane	α-HCH	291.0	38,295.0	4.0	1.00	4.91	340.11	9.24
Hexachlorocyclohexene-d	HexaCX-d	289.0	16.8	0.0	0.00	0.00	0.00	0.00
β-hexachlorocyclohexane	β-HCH	291.0	0.4	0.0	0.11	0.54	11.53	0.31
γ-HCH (Lindane)	γ-HCH	291.0	126,985.3	13.2	3.00	14.73	958.44	26.03
Heptachlorocyclohexane-1	HeptaCH-1	325.0	106,325.6	11.0	0.00	0.00	551.88	14.99
δ-Hexachlorocyclohexane	δ-HCH	291.0	71,041.2	7.4	1.20	5.89	610.32	16.58
ε-Hexachlorocyclohexane	ε-HCH	291.0	16,327.8	1.7	0.50	2.45	115.00	3.12
Heptachlorocyclohexane-2	HeptaCH-2	325.0	40,659.8	4.2	0.20	0.98	319.79	8.69
Heptachlorocyclohexane-3	HeptaCH-3	325.0	19,245.1	2.0	0.10	0.49	123.92	3.37
Total			964,059.3	100	20.37	100.00	3681.87	100.00

**Table 2:** Composition and toxicity of the aqueous elutriates obtained at neutral conditions from soils B1 and B2 ($\frac{V_{L}}{W}=2\frac{L}{\mathrm{kg}}$) and alkaline conditions in soil B2 ($\frac{V_{L}}{W}=0.4\frac{L}{\mathrm{kg}}$). The effective nominal concentration of identified COCs is also included.

Acronym	$EC_{50}{}_{j}$ (mg/L)	C _B1 aq_ (mg/L)	C _B2 aq_ (mg/L)	C_B2_ Pv Alkaline Pretreatment (mg/L)
CB	11.30	0.00	0.00	0.00
1,3-DCB	5.10	0.00	0.06	0.05
1,4-DCB	4.50	0.00	0.43	0.04
1,2-DCB	4.05	0.00	0.41	0.23
1,3,5-TCB	3.44	0.00	0.03	0.14
1,2,4-TCB	3.44	0.00	1.97	6.96
1,2,3-TCB	0.82	0.00	0.12	1.27
TetraCB-a	0.61	0.00	0.24	0.28
TetraCB-b	0.61	0.01	0.35	0.49
γ-PentaCX	-	0.00	0.85	0.00
PCB	-	0.00	0.00	0.01
δ-PentaCX	-	0.01	7.10	0.00
θ-PentaCX	-	0.00	0.79	0.00
HexaCX-a	-	0.00	0.20	0.00
β-PentaCX	-	0.00	2.48	0.00
η-Penta CX	-	0.00	0.29	0.00
HexaCX-b	-	0.00	0.01	0.00
HexaCX-c	-	0.00	1.32	0.00
α-HCH	-	0.00	3.14	0.00
HexaCX-d	-	0.00	0.00	0.00
β-HCH	-	0.00	0.65	0.00
γ-HCH	2.03	0.03	7.79	0.00
HeptaCH-1	-	0.01	2.24	0.00
δ-HCH	3.50	0.02	15.15	0.00
ε-HCH	-	0.00	7.29	0.00
HeptaCH-2	**-**	0.00	2.03	0.00
HeptaCH-3	**-**	0.00	1.94	0.00
Total COCs		0.06	56.89	9.47
TUs exp Equation (6)		Non-detected	17.24	5.72
TUs estim Equation (8)		0.03	10.07	4.95

**Table 3:** COCs remaining in the soil columns after injection of 8 Pvs of reagents and 8 Pvs of tap water.

Acronym	C_j_ (mg/kg)	C_j_ (mg/kg)	C_j_ (mg/kg)
	T1 (ISCO)	T2 (S-ISCO-5)	T3 (S-ISCO-10)
CB	0.00	0.00	0.00
1,3-DCB	0.00	0.14	0.03
1,4-DCB	0.00	0.00	0.05
1,2-DCB	0.00	1.27	1.30
1,3,5-TCB	3.52	0.55	0.56
1,2,4-TCB	98.06	6.92	6.65
1,2,3-TCB	17.39	1.39	1.21
TetraCB-a	36.41	10.15	9.06
TetraCB-b	66.06	12.19	17.25
γ-PentaCX	0.00	12.91	20.63
PCB	3.00	2.1	2.03
δ-PentaCX	0.00	0.00	0.00
θ-PentaCX	0.00	0.00	0.00
HexaCX-a	0.00	0.00	0.00
β-PentaCX	0.00	0.00	0.00
η-Penta CX	0.00	0.00	0.00
HexaCX-b	0.00	0.00	0.00
HexaCX-c	0.00	0.00	0.00
α-HCH	0.00	0.00	0.00
HexaCX-d	0.00	0.00	0.00
β-HCH	0.00	0.00	0.00
γ-HCH	0.00	0.00	0.00
HeptaCH-1	0.00	0.00	0.00
δ-HCH	0.00	0.00	0.00
ε-HCH	0.00	0.00	0.20
HeptaCH-2	0.00	0.00	0.00
HeptaCH-3	0.00	0.00	0.00
Total COCs	224.43	47.62	62.08

**Table 4:** Mass balance of COCs in T1, T2, and T3 columns.

	Units	T1 (ISCO)	T2 (S-ISCO 5)	T3 (S-ISCO 10)
Final COCs soil	mg/kg	224.43	56.23	62.3
COCs in gathered aqueous phases from flushed Pvs with reagents (PS)	mg/L	1.26	4.3	28.46
COCs in gathered aqueous phases from flushed Pvs with tap water	mg/L	2.32	5.2	28.4
Mass of COCs in treated soil	mg	11.22	2.81	3.1
Mass of COCs in gathered Pvs flushed with reagents	mg	0.10	0.34	2.28
Mass of COCs in gathered aqueous phases from flushed Pvs with tap water	mg	0.19	0.42	2.27
Initial mass of COCs in soil (pH = 7)	mg	184.05	184.05	184.05
Initial mass of COCs in soil (pH = 12)	mg	117.05	117.05	117.05
X_COCs_		0.90	0.97	0.94

**Table 5:** Experimental conditions in column runs. The flow rate used in all Pv injections was 0.3 mL/min.

Soil Columns	T1	T2	T3
	ISCO	S-ISCO	S-ISCO
Initial COCs (mg/kg)	3682	3682	3682
Soil height (cm)	4.7	4.7	4.7
Soil mass (g)	53	51	52
Pv (mL)	11.2	10.7	10.9
**Alkaline Pretreatment**	T1	T2	T3
C_NaOH_ (mM)	210	210	210
Pvs injected	2	2	2
Time between next Pv injection (h)	24	24	24
**Reagents injected**	T1	T2	T3
C_surf_ (g/L)	0	5	10
C_PS_ (mM)	210	210	210
C_NaOH_ (mM)	210	210	210
Pvs injected	8	8	8
Time between next Pv injection (h)	96	96	96
**Washing with tap water**	T1	T2	T3
Pvs injected	8	8	8
Time between next Pv injection (h)	0.5	0.5	0.5

## Supplementary Materials

The following supporting information can be downloaded at: https://www.mdpi.com/article/10.3390/molecules27248965/s1, Table S1. PS (mM) flushed at each Pv; Table S2 Composition of the 8 gathered Pvs flushed from columns T1, T2 and T3 96 h after the last Pv was eluted.
