# Supplementary material for: Acute Toxicity Evaluation of Lindane-Waste Contaminated Soils Treated by Surfactant-Enhanced ISCO
Source: Molecules. 2022 Dec 16;27(24):8965. doi: 10.3390/molecules27248965 (PMC9786798; doi:10.3390/molecules27248965)
Supplement: Supplementary file 1 [file molecules-27-08965-s001.zip › molecules-2100999-supplementary.pdf]

Acute toxicity evaluation of lindane-waste contaminated soils treated by surfactant-enhanced ISCO

Aurora Santos*, Raúl García-Cervilla, Alicia Checa-Fernández, Carmen Domínguez, David Lorenzo

Dpto. Ingeniería Química y de Materiales, Facultad de Ciencias Químicas, Universidad Complutense Madrid. Ciudad Universitaria S/N. 28040, Madrid, Spain

Paper submitted to

Molecules

for consideration

Special Issue "Residues of Organic Pollutants in Environmental Samples"

Supplementary Material

Table S-1. PS (mM) flushed at each Pv

Pv	ISCO	S-ISCO 5	S-ISCO 10
	PS	PS	PS
**1**	31.7	19.2	17.2
**2**	53.5	25.9	18.1
**3**	60.7	36.2	26.0
**4**	85.1	71.7	42.6
**5**	110.0	110.0	76.0
**6**	130.2	125.0	82.0
**7**	170.0	130.1	90.2
**8**	200.0	145.2	98.1

Table S-2 Composition of the 8 gathered Pvs flushed from columns T1, T2 and T3 96 h after the last Pv was eluted

Acronym	C_j (mg/L)	C_j (mg/L)	C_j (mg/L)
	Σ8 PVs flushed from T1 (32 days)	Σ8 PVs flushed from T2 (32 days)	Σ8 PVs flushed from T3 (32 days)
CB	0.00	0.00	0.00
1,3-DCB	0.00	0.00	0.03
1,4-DCB	0.04	0.00	0.01
1,2-DCB	0.03	0.00	0.07
1,3,5-TCB	0.02	0.07	0.71
1,2,4-TCB	0.84	1.85	18.00
1,2,3-TCB	0.12	0.43	3.20
TetraCB-a	0.08	0.69	2.10
TetraCB-b	0.10	1.21	3.20
γ-PentaCX	0.00	0.03	0.50
PCB	0.00	0.01	0.64
δ-PentaCX	0.03	0.00	0.00
θ-PentaCX	0.00	0.00	0.00
HexaCX-a	0.00	0.00	0.00
β-PentaCX	0.00	0.00	0.00
η-Penta CX	0.00	0.00	0.00
HexaCX-b	0.00	0.00	0.00
HexaCX-c	0.00	0.00	0.00
α-HCH	0.00	0.00	0.00
HexaCX-d	0.00	0.00	0.00
β-HCH	0.00	0.00	0.00
γ-HCH	0.00	0.00	0.00
HeptaCH-1	0.00	0.00	0.00
δ-HCH	0.00	0.00	0.00
ε-HCH	0.00	0.00	0.00
HeptaCH-2	0.00	0.00	0.00
HeptaCH-3	0.00	0.00	0.00
Total COCs	1.26	4.30	28.46
PS (mM)	95	86	71
Csurf average (g/L)	0	0.13	0.22
TUs exp **Eq. (5)**	N.D	5.6	22.1
TUs estim. **Eq. (7)**	N.D	4.2	18.3
